# Recommendations for POCUS Curriculum in Canadian Undergraduate Medical Education: Consensus from the Inaugural Seguin Canadian POCUS Education Conference

**DOI:** 10.24908/pocus.v8i1.16153

**Published:** 2023-04-26

**Authors:** Sacha Weill, Daniel Armand Picard, Daniel J Kim, Michael Y Woo

**Affiliations:** 1 Faculty of Medicine, University of Ottawa Ottawa, Ontario Canada; 2 Department of Emergency Medicine, University of British Columbia & Vancouver General Hospital Vancouver, British Columbia Canada; 3 Clinical Epidemiology Program, Ottawa Hospital Research Institute Ontario Canada

**Keywords:** POCUS, Curricular Innovation, Recommendations, Medical Student

## Abstract

Point of care ultrasound (POCUS) in Canadian undergraduate medical education (UGME) is limited. To address this paucity, the inaugural Seguin Canadian POCUS Education Conference hosted 14 of the 17 Canadian medical schools to develop a list of recommendations for POCUS education in Canadian UGME. Attending schools were divided into delegations consisting of a pre-clerkship student, a clerkship student and a staff physician. Recommendations were developed via a modified consensus development panel. Delegations submitted school-specific POCUS education summary documents for roundtable discussions, which yielded an initial set of recommendations. These were then summarized in a large group setting and voted upon for adoption with an a priori agreement threshold of 80%. Conference attendees developed 14 recommendations which 87% of participants agreed to adopt. Conference recommendations reflect the opinions of Canadian trainees and POCUS education experts thus serving as a framework for UGME POCUS education in Canada.

## Introduction

Point of care ultrasound (POCUS) is a targeted ultrasound examination performed by the clinician at the bedside. Alongside the history and physical examination, POCUS allows the clinician to complete a more diagnostically comprehensive evaluation 1.The effectiveness of the modality is well documented in the literature; its use is associated with improved safety outcomes, reduced costs and decreased need for consultative diagnostic imaging 1.Unsurprisingly, this had led to greater desire for curricular implementation of POCUS in undergraduate medical education (UGME) from students, faculty and administration alike 2, 3, 4. 

Despite this enthusiasm, POCUS teaching across Canada is variable and often insufficient. This is evidenced by a 2014 national survey of UGME POCUS education which demonstrated that only 50% of Canadian medical schools had integrated some form of ultrasound teaching into their curriculum. In those offering ultrasound teaching, the majority only offered 1-5 hours of instruction annually 2.This does not reflect clinical practice, where many specialties integrate basic POCUS skills into patient care 5, 6, 7, 8, 9, 10.

This mismatch between UGME and clinical experience prompted the creation of the Seguin Canadian POCUS Education Conference (SCPEC), which hosted delegates from 14 out of the 17 Canadian medical schools on August 14, 2021. The objective of this conference was to develop recommendations for POCUS education implementation in Canadian UGME with the goal of better preparing medical students for POCUS-integrated clinical practice.

## Methods

We used a modified consensus development panel to establish consensus and generate recommendations. Participants were recruited through emails submitted to all 17 Canadian medical school’s student POCUS or emergency medicine interest groups, to identify pre-clerkship and clerkship delegates. The delegates were then asked to recruit a staff physician with POCUS expertise to join their delegation. Staff physicians with POCUS expertise were also contacted directly. Thirty-nine participants from 14 Canadian medical schools ultimately attended (Table 1). Three medical schools (l’Université de Laval, l’Université de Sherbrooke and the University of Manitoba) did not attend.

**Table 1:** Attending medical school and participant breakdown for SCPEC 2021.

**School**	**Pre-Clerkship Delegates**	**Clerkship Delegates**	**Physician Delegates and Specialty**	**Delegates per School**
Western University	0	2	1 (EM)	3
University of Toronto	0	2	1 (EM)	3
McMaster University	1	1	1 (EM)	3
Queen's University	1	1	1 (EM)	3
University of Ottawa	1	1	1 (EM)	3
Northern Ontario School of Medicine	1	1	0	2
McGill University	1	1	1 (EM)	3
Université de Montréal	1	1	0	2
Memorial University	1	1	1 (EM)	3
Dalhousie University	1	1	0	2
University of Alberta	1	1	1 (EM)	3
University of British Columbia	1	1	3 (EM)	5
University of Saskatchewan	1	1	1 (EM)	3
University of Calgary	0	0	1 (IM)	1
Total	11	15	13	39

Topics for discussion were initially sourced from a 2018 scoping review of UGME ultrasound education. Subjects included curriculum structure, teaching, adjunct teaching methods, barriers, and solutions 11.Prior to the conference, attendees were asked to highlight their school’s current POCUS education program based on these topics to form school specific summary documents. From this information, the following list of topics was chosen for inclusion by conference leaders: principles of POCUS education, barriers to its integration, and solutions to these barriers.

The conference took place virtually on August 14, 2021, using Zoom (Zoom Video Communications Inc, San Jose, CA). Delegations were divided into groups of three schools. These small groups discussed the topics generated prior to the conference, creating an initial set of recommendations per group. All participants then joined a conference-wide roundtable and adapted their initial work into a final list of recommendations. Subsequently, attendees voted to endorse these recommendations using Zoom’s reaction system to vote for, against or abstain (by not submitting a reaction). We established an a priori consensus threshold of 80%, similar to other consensus-building initiatives 12, 13.

## Results

Conference attendees generated a list of 14 recommendations, grouped under the themes of POCUS education priorities, barriers to implementation, and solutions to barriers. These recommendations are listed in Table 2. Voting resulted in a clear consensus with 87% (34 of 39) endorsing the recommendations in their entirety and agreeing to promote them locally and nationally; 5 individuals (13%) abstained from the vote.

**Table 2:** SCPEC 2021 recommendations list.

**Education**
1. POCUS education should start early 2. POCUS should be taught longitudinally 3. Prioritize teaching ultrasound basics and anatomy in pre-clerkship and applied scanning with clinical correlation in clerkship 4. Create a POCUS elective in clerkship for learners desiring more advanced ultrasound proficiency 5. Establish that medical students should be familiar and comfortable with POCUS, not necessarily experts
**Barriers**
6. Acknowledge that there is a lack of space for POCUS education in a UGME curriculum 7. Identify that there is lack of ultrasound machines dedicated for teaching 8. Acknowledge that there is a lack of impetus from some administration to formally integrate POCUS into UGME 9. Recognize that there is a lack of qualified instructors to teach POCUS
**Solutions**
10. Use providers outside of emergency medicine to teach POCUS 11. Utilize near peer instruction (NPI) to address the lack of qualified instructors 12. Leverage a flipped classroom model and more online educational resources to allow for more hands-on POCUS instruction 13. Explore options for handheld device acquisition and industry partnership to save on costs and materials 14. Appreciate the role of student POCUS interest groups in education and support them

## Discussion

The goal of SCPEC was to generate consensus around a list of recommendations from a group of student leaders and physicians on POCUS education in Canadian UGME. This initiative is uniquely different from other POCUS education consensus efforts as it was primarily driven by medical student stakeholders, rather than faculty experts 14, 15. For example, in Hoppman et al.’s international consensus initiative, students were only implicated in the early stages of consensus iteration and had their work modified by consultants. Despite this, many of their recommendations paralleled SCPEC’s 14. Notably, their group endorsed longitudinal learning that complements the curricular structure of medical school. Similarly, they emphasized that curricular content should be appropriate for the medical student level, focusing on the basics, but also be applicable to all students, regardless of specialty choice 14.

From a Canadian perspective, Ma et al. published a faculty-led consensus document outlining important curricular POCUS elements to be included in UGME 15. Our suggestions mirror many of those outlined by Ma et al. They similarly endorsed the use of near-peer teaching models, interprofessional teams of instructors beyond emergency medicine, simulation, and novel educational means such as videos or podcasts 15. 

Although novel in the realm of POCUS, a similar student-led consensus generating initiative was completed by Chicorelli et al. 16 regarding interdisciplinary education. Their methodology was similar to ours in that their initiative was student-driven, faculty supported and utilized a progressive consensus forming methodology where they used breakout rooms followed by a roundtable discussion to establish consensus. 

Given this high level of fidelity with previous initiatives, our student-led effort can complement existing recommendations, aiding in advocacy for POCUS education at the national and individual medical faculty level. 

### Education

#### POCUS education should start early on. 

Early instruction promotes familiarity with ultrasound concepts and allows students to develop competencies naturally, rather than learn about the modality later in clinical practice with little foundational knowledge. 

#### POCUS should be taught longitudinally. 

There is a clear need for longitudinal learning to ensure long-term retention of skills. Simply learning an ultrasound technique once, without opportunities to continually practice, is not sufficient. The absence of longitudinal teaching through repeated sessions greatly reduces learner competency and comfort 17, 18. An evaluation of spaced repetition and longitudinal access to learning at the McGill University and American medical schools have demonstrated improved knowledge acquisition and retention 11, 19. 

#### Prioritize teaching ultrasound basics/anatomy in pre-clerkship and applied scanning with clinical correlation in clerkship. 

A POCUS curriculum should mirror the typical progression of medical school 18, 20, 21.In practice, this means teaching ultrasound basics/anatomy in pre-clerkship and applied scanning with clinical correlation in clerkship 22.Canadian and American POCUS education experts have used this principle to prioritize what POCUS concepts are essential in UGME 15, 18. Kumar et al.’s review of cardiac POCUS education in UGME highlighted the importance of this concept. Elicited themes included using POCUS as a tool to teach anatomy, to enhance physical examination, and to prepare medical students towards further differentiation of POCUS skills in their clerkship years and with postgraduate specialty-specific training 23.

#### Create a POCUS elective in clerkship for learners desiring more advanced ultrasound proficiency. 

A pathway for students wanting to further develop their skills, beyond general POCUS competency, should be offered to encourage and reward their interest. A POCUS elective can satisfy this need. In fact, this kind of elective is successfully being implemented with a high level of engagement at the University of Saskatchewan, Northern Ontario School of Medicine, Memorial University of Newfoundland, the University of Manitoba, and soon the University of British Columbia. 

#### Establish that medical students should be familiar and comfortable with POCUS, not necessarily experts. 

Complete expertise in ultrasonography should not be the expectation of undergraduate medical learners. Rather an understanding and familiarity with image interpretation, ultrasound physics and comfort with probe handling are more realistic goals.

Learner evaluation is often scored on a 1-5 entrustment scale, whereby a score of 1 indicates that a supervisor must take over the task, and 5 when a student can complete the task independently without supervision 24. For POCUS, this would mean a student can complete an ultrasound study with minimal prompting or supervision, corresponding to an entrustment score of 3. 

### Barriers

#### Acknowledge that there is a lack of space for POCUS education in a UGME curriculum. 

A widely touted barrier to greater POCUS instruction is a lack of space in UGME curricula. Current hallmarks of education, such as anatomy, pathology and traditional physical examination, are gauged to be more relevant, supposedly superseding the educational value of POCUS. However, POCUS can complement these classical disciplines, and even replace outdated physical exam skills 25. 

#### Identify that there is lack of ultrasound machines dedicated for teaching. 

Another limiting factor for POCUS integration in UGME is a lack of machines available for teaching. These machines are an expensive initial investment and it can be difficult to convince administrators to purchase them, while managing an already strained budget. Although ultrasound machines may be available in environments such as simulation centers, access is often not reliable enough for sustained and longitudinal education. 

#### Acknowledge that there is a lack of impetus from some administration to formally integrate POCUS into UGME. 

As evidenced by their incredible involvement in the SCPEC conference, students are motivated to learn POCUS. Schools that have integrated POCUS have received overwhelmingly positive feedback from students 2, 3, 4. In fact, a review of the literature of POCUS integration in UGME found that many students exposed to ultrasound chose to pursue residency programs with a stronger ultrasound component 11. Despite student and faculty engagement, insufficient teaching stipends, a lack of administrative support and limited protected teaching time for clinician instructors, limit student exposure to POCUS. Given these barriers, students and physician leaders often resort to extracurricular education, such as interest groups or self-learning, to supplement POCUS instruction.

#### Recognize that there is a lack of qualified instructors to teach POCUS. 

A common barrier for the introduction of POCUS in UGME is the lack of qualified instructors that are available to give timely and longitudinal teaching to learners. As POCUS is most commonplace in emergency medicine, many programs limit their ultrasound education to emergency medicine applications and primarily use emergency physicians as instructors 11.

### Solutions

#### Use providers outside of emergency medicine to teach POCUS. 

POCUS is not exclusive to emergency medicine but is used routinely by multiple specialties. Although not an exhaustive list, traditional comprehensive imaging specialists like cardiologists, radiologists and obstetrician-gynecologists, as well as other POCUS users like intensivists, internists, anesthesiologists, and pediatricians to name only a few, can all serve as instructors. In fact, successful ultrasound programs have capitalized on this rich supply of practitioners 11. Furthermore, skilled non-physician medical professionals who use POCUS, such as sonographers and anatomists, could be a great source of instruction for students. Indeed, certain American programs are successfully utilizing these professionals as instructors 26, 27. Additionally, involving standardized patients with POCUS knowledge can be another excellent source of POCUS learning 28.

#### Utilize near peer instruction (NPI) to address the lack of instructors. 

To further address the lack of instructors, SCPEC attendees recommended using near-peer instruction (NPI), whereby medical students who have a special interest or training in POCUS are able to instruct their peers. Schools capitalizing on NPI have been able to reduce the teaching load placed on faculty 29, 30, 31, 32, 33.For example, Hendriksz et al. demonstrated that only 2 hours of faculty time was required to train a peer mentor to an acceptable level of expertise for a particular teaching session 29. Similarly, a randomized controlled trial showed that student-mentors, trained with short teaching intervals and self-directed learning, could be taught to instruct musculoskeletal ultrasound at a level similar to faculty 34. As an added benefit, student-tutors profit from this teaching method; peer-teaching prepares them for a mentoring role that will be expected during residency and serves to consolidate their own knowledge 31.

#### Leverage a flipped classroom model and more online educational resources to allow for more hands-on POCUS instruction. 

Prioritizing a flipped classroom model in POCUS instruction can help address the lack of space in curricula; maximizing theoretical learning online can leave more time for hands-on, in-class instruction. Fuchs et al. showed that online learning can be just as effective as traditional bedside POCUS instruction. Specifically, students learning cardiac ultrasound through an eLearning platform and independent practice with an ultrasound mannequin performed just as well as students receiving in-person instruction for the same scans 35. 

#### Explore options for handheld device acquisition and industry partnership to save on costs and materials. 

Partnerships with ultrasound companies can tackle the lack of access to ultrasound equipment and are already being successfully utilized in schools with dedicated UGME POCUS curricula 36. While industry partnerships are important to reduce the economic burden of introducing POCUS, it was the attendees’ beliefs that learning content must remain independent of industry influence. Additionally, handheld models can circumvent high upfront costs of traditional ultrasound machines. These tools exist at a fraction of the cost and their portability lends them better to a dynamic learning environment 37.

#### Appreciate the role of student POCUS interest groups in education and support them. 

As was evidenced by SCPEC, medical students are at the forefront of POCUS education and advocacy. This interest has been manifested through the various POCUS student interest groups across Canada. These groups not only create excitement around POCUS, but also provide a significant amount of POCUS teaching and act as auxiliary instructors.

## Conclusion and Next Steps

The inaugural Seguin Canadian POCUS Education Conference was able to create consensus derived recommendations, paving the way for the promotion of POCUS education in medical schools across Canada. To ensure success, a longitudinal and nationally coordinated approach is key. Thus, a national student POCUS society was created through SCPEC and future efforts to entrench the conference and its goals, through institutional bodies, such as the Association of Faculties of Medicine of Canada, are underway. While this iteration of the conference aimed to generate consensus around a list of recommendations for POCUS education, future topics could include innovations in POCUS education such as gamification theory, remote learning and integration of hand-held POCUS machines. This progression is already underway with the second iteration of SCPEC occurring successfully in the summer of 2022. In summary, SCPEC was a student-led initiative for the advancement of UGME POCUS education in Canada.

## Statement of ethics approval/consent

The University of Ottawa’s Research Ethics Board (REB) deemed REB approval for this project to be unnecessary as it fell within the scope of quality improvement and program evaluation.

## Disclosures

DJK provides consultant services to Fujifilm Sonosite. DJK and MW are both members of the Canadian Ultrasound Consensus for Undergraduate Medical Education Committee, and of the Canadian Association of Emergency Physicians Ultrasound Committee. 
